# *Adenanthera pavonina*, a potential plant-based protein resource: Seed protein composition and immunohistochemical localization of trypsin inhibitors

**DOI:** 10.1016/j.fochx.2022.100253

**Published:** 2022-02-12

**Authors:** Hari B. Krishnan, Sunhyung Kim, Adriano E. Pereira, Alexander Jurkevich, Bruce E. Hibbard

**Affiliations:** a Plant Genetics Research Unit, USDA, Agricultural Research Service, Columbia, MO 65211, USA; b Division of Plant Science and Technology, University of Missouri, Columbia, MO 65211, USA; c Advanced Light Microscopy Core, Christopher S. Bond Life Sciences Center, University of Missouri, Columbia, MO 65211, USA

**Keywords:** *Adenanthera*, Confocal microscopy, Immunocytochemistry, Trypsin inhibitor, Red bead tree, Western corn rootworm

## Abstract

*Adenanthera pavonina*, an underutilized tropical tree, is being promoted as an alternative food source for meeting the nutritional needs of human and animals. In this study, we have shown that trypsin inhibitors as one of the predominant proteins in the seeds of *A. pavonina*. DE-52 column chromatography resulted in the identification of four peaks with trypsin inhibitor activity. SDS-PAGE and immunoblot analyses revealed DE-52 peaks A and B were enriched in 17 and 15 kDa proteins and these proteins cross-reacted against soybean trypsin inhibitor antibodies. Simulated gastric fluid digestion revealed that the 15–17 kDa proteins are resistant to pepsin digestion. Roasting the seeds lowered the trypsin inhibitor activity while boiling intact seeds elevated the enzyme activity. However, the trypsin inhibitor activity was completely abolished when the seeds were boiled without their seed coats. Immunohistochemical detection and confocal microscopy demonstrated that trypsin inhibitors were localized in the cell cytosol.

## Highlights

• Trypsin inhibitors are abundant in the seeds of *Adenanthera pavonina.*
• *A. pavonina* trypsin inhibitors cross react with soybean trypsin inhibitor antibodies.
• Boiling *A. pavonina* seeds inactivates the trypsin inhibitors.
• *A. pavonina* trypsin inhibitors are resistant to pepsin digestion.
• *A. pavonina* trypsin inhibitors are localized in the cell cytosol.

## 1 Introduction

*Adenanthera pavonina* is a fast-growing legume tree that belongs to the subfamily *Mimosoideae*. This tree is also known as red bead tree, red sandalwood, saga, and coral tree and is cultivated for forage and soil improvement. *Adenanthera pavonina* is endemic to India and Southeast China and later introduced throughout the tropics. Importantly, *A. pavonina* has been widely used in traditional medicine to treat a range of diseases including hypertension, diarrhea, gout, rheumatism, and cancer (Geronco et al., 2020). The leaves, bark, and seeds of this tree contain a wide array of chemical compounds including flavonoids, alkaloids, steroids, saponins, polyphenols, triterpenoids, coumarins and glycosides. Pharmacological studies have demonstrated antinociceptive, cytoprotective, anti-inflammatory, antihyperglycemic and hypolipidemic effects of leaf and seed extracts (Ara et al., 2010, Pandhare et al., 2012a, Pandhare et al., 2012b). A recent review article has highlighted the therapeutic and technological potential of *A. pavonina* (Geronco et al., 2020). Importantly, many studies have demonstrated that protease inhibitors extracted from *A. pavonina* seeds could play an insecticidal function in the control of phytophagous insects (Macedo et al., 2002, Rodrigues Macedo et al., 2003, Macedo et al., 2010; Silva, Freire, Parra, Marangoni, & Macedo, 2012; Sasaki, Jacobowski, de Souza, Cardoso, Franco & Macedo, 2015).

In addition to its insecticidal and therapeutic potential *A. pavonina* is also being considered as alternative food source especially in developing countries. Due to population pressure, low agricultural production, and food shortages, researchers are promoting the use of underutilized plant sources for meeting the nutritional needs of human and animals. The seeds and young leaves of *A. pavonina* are consumed in some Asian and African countries. The proximate composition of *A. pavonina* seeds have been investigated and was found to contain a high percentage of protein, oil, and carbohydrates. The protein content of the seed ranges from 22 to 31% while that of oil from 11 to 13% (Kitumbe et al., 2013, Sultana and Gulzar, 2012). The fatty acid profile of the seed revealed that linoleic acid was the most abundant followed by oleic acid, palmitic acid and gadoleic acid. In addition, the seed was also found to be rich in lignoceric acid, an essential fatty acid for the growth, development, and maintenance of the brain (Nwafor, Egonu, Nweze, & Ohabuenyi, 2017). *A. pavonina* seed oil, due to its high nutritional value, can be used as an alternative source of edible oil for humans and animals.

Preliminarily studies have shown that the seeds of *A. pavonina*, like other legumes, is rich in protein (Nwafor et al., 2017). The protein content of *A. pavonina* seeds was reported to be comparable to that of other widely consumed legumes such as soybean and cowpea. In several regions of Southeast Asia and the South Pacific, cooked or roasted *A.* seeds are eaten like peanuts (Whistler, 2000). In Samoa, eating either the raw or roasted seeds of *A. pavonina* is being promoted as means to reduce the negative effects of obesity (Huml et al., 2020). Processing (roasting and boiling) of the seeds was shown to improve the palatability and nutritional value of *A.* seeds by lowering the endogenous anti-nutritional components. Due to the high nutritional value, it was proposed that processed seeds of *A. pavonina* can be exploited as an alternative source of nutrients to reduce malnutrition in developing countries (Nwafor et al., 2017). Recent studies have examined the physiochemical, structural, nutritional, and biochemical qualities of pro-milk extract derived from the seeds of *A. pavonina* (Afolabi et al., 2018, Dwitanti et al., 2020). Interestingly, this study found pro-milk from *A. pavonina* had significantly higher level of protein and mineral content when compared to soy milk. It was suggested that *A. pavonina* pro-milk may offer benefits against neurological diseases and promote general health (Afolabi et al., 2018). Despite its nutritional potential in combating malnutrition and food security only limited biochemical studies have been conducted on *A. pavonina* seed proteins. We hypothesized that biochemical characterization of the seed proteins of this underutilized legume will provide a better understanding of its nutritive potential.

In this study, we have partially purified some of the abundant seed proteins of *A. pavonina* and subjected them to biochemical and immunological characterization. Additionally, we also examined the effect of trypsin inhibitors extracted from *A. pavonina* seeds on western corn rootworm (*Diabrotica virgifera* LeConte (Coleoptera: Chrysomelidae) larvae, a devastating corn insect pest in the United States.

## 2 Materials and methods

### 2.1 Seed material

Seeds of *Adenanthera pavonina* (Accessions PI 9416 and PI 105707) were obtained from the USDA-ARS GRIN Global collection.

### 2.2 Insects

The WCR beetles were collected in Hart (Castro Co.), Texas, in the summer of 2018 and sent to the Plant Genetics Research Unit greenhouse, USDA/ARS, in Columbia, MO. The diapausing beetles were placed in BugDorm cages (30 cm^3^) and provided with 15% agar (water source), dry artificial diet (Frontier Agriculture Sciences, Newark, DE), and zucchini (*Cucurbita pepo* L.) slices. For oviposition, 9-cm petri dishes were 1/2 filled with 80-mesh sifted moist soil with grooves. The F1 beetles were separated by sex allowed to mate with corresponding non-diapausing beetles from USDA/ARS in Brookings, SD, to break diapause. Western corn rootworm rearing techniques are similar as described earlier (Meihls et al., 2008).

### 2.3 Protein extraction

Mature dry seeds were first frozen in liquid nitrogen and ground to a fine power with a mortar and pestle. To isolate total protein, 30 mg of finely ground *A. pavonina* seed powder was transferred to a 2 mL Eppendorf tube. To this tube, 1 mL of sodium dodecyl sulfate (SDS)-sample buffer (60 mM Tris-HCl, pH 6.8, 2% SDS (w/v), 10% glycerol (v/v), and 5% 2-mercaptoethanol (v/v) was added. Contents of the tube were vortexed for 10 min at room temperature followed by boiling at 100° C for 5 min. Residual materials were removed by centrifugation at 15800xg for 5 min and the resulting supernatant was transferred to a new 1.5 mL plastic tube and designated as total seed protein fraction.

Sequential extraction of albumin, globulin, prolamin, and glutelin fractions were performed as described earlier (Krishnan, White, & Pueppke, 1992). Briefly, 30 mg of *A. pavonina* seed powder was extracted with 1 mL of 10 mM Tris-HCl, pH 6.8, 1 mM EDTA by vigorously mixing the contents followed by centrifugation at 15800xg for 5 min. The clear supernatant was transferred to a new plastic tube and designated as albumin fraction. To the pellet fraction, 1 mL of 10 mM Tris-HCl, pH 6.8, 1 mM EDTA and 500 mM NaCl was added. The content of the tube was again vigorously mixed and subjected to centrifugation as stated above. The resulting supernatant was designated as globulin fraction. The pellet resulting from the centrifugation step was extracted with 1 mL of 50% isopropanol and 2.5% 2-mercaptoethanol on a vortexer for 10 min. Following centrifugation at 15800xg for 5 min, the supernatant was saved and designated as prolamin fraction. The residual pellet was extracted with 1 mL of 60 mM Tris-HCl, pH 6.8, 2% SDS (w/v), 10% glycerol (v/v), and 5% 2-mercaptoethanol. Contents of the tube were vortexed for 10 min at room temperature followed by boiling at 100° C for 5 min. Residual materials were removed by centrifugation at 15800xg for 5 min and the resulting supernatant was designated as glutenin fraction.

### 2.4 1-D electrophoresis

*Adenanthera pavonina* seed proteins were resolved by 13.5% SDS-PAGE gels using a Hoeffer SE 250 Mini-Vertical electrophoresis apparatus (GE Healthcare, Pittsburg, PA, U.S.A.). Separation of proteins was attained with a constant 20 mA/gel until the tracking dye bromophenol blue reached the bottom of the gel. Following electrophoresis, the gels were stained with Coomassie Blue R-250 solution overnight.

### 2.5 Immunoblot analysis

Seed proteins of *A. pavonina* were first resolved on SDS-PAGE gels as described above. Separated proteins were transferred to nitrocellulose membranes with the aid of Bio-Rad Trans-Blot Turbo Transfer system. After transfer of proteins, the membranes were incubated with TBS (10 mM Tris-HCl, pH 7.5, 500 mM NaCl) containing 5% non-fat dry milk for 1 h at room temperature to prevent non-specific binding of antibodies. Following this step, the nitrocellulose membranes were incubated over-night with antibodies raised against purified soybean trypsin inhibitor (Krishnan, 2001) that had been diluted 1:5,000 in TBST (TBS with 3% non-fat dry milk containing 0.2% Tween 20). Subsequently, the membrane was washed three times with TBST and incubated with goat anti-rabbit IgG-horseradish peroxidase conjugate that had been diluted 1:5,000 in TBST. Proteins reacting specifically with the soybean trypsin inhibitor antibodies were detected using the SuperSignal West Pico kit (Pierce, Rockford, IL, U.S.A.).

### 2.6 2-D electrophoresis

Seed protein preparation and their subsequent separation by 2-D electrophoresis was conducted following established protocols (Krishnan, Natarajan, Mahmoud, & Nelson, 2007). For isoelectric focus (IEF) 300 µg of protein sample or 100 µg of DE-52 fractions were loaded on IEF strips using in-gel rehydration and linear gradient, 13 cm IPG strips (GE Healthcare, Pittsburg, PA, U.S.A.). Following IEF and SDS-PAGE, the gels were fixed in 5:4:1 (methanol:water:acetic acid) for 30 min, briefly rinsed in water, and stained in a Coomassie G-250 solution for overnight. Coomassie stained gels were scanned using an Epson V700 Perfection scanner controlled through Adobe Photoshop.

### 2.7 DEAE anion-exchange chromatographic separation of *A. pavonina* trypsin inhibitors

Five grams of *A. pavonina* seed powder was extracted with 100 mL of 20 mM Tris-HCl, pH 8.0 in 250 mL flask on a magnetic stirrer for about 15 min. The slurry was clarified by centrifugation at 13320g for 15 min. To the clear supernatant CaCl_2_ was added to a final concentration of 10 mM and incubated at room temperature for 10 min followed by centrifugation as stated above. To the resulting supernatant solid ammonium sulfate was gradually added to 45% saturation and left on ice for 30 min. Precipitated proteins were discarded. To the supernatant solid ammonium sulfate was slowly added to 90% saturation. After incubation on ice overnight the precipitated proteins were recovered by centrifugation at 13320g for 15 min. The pellet (40–90% salt cut) was dissolved in 20 mL of 100 mM Tris-HCl, pH 8.0 and dialyzed against 2 L of 100 mM Tris-HCl, pH 8.0 at 4 °C.

The dialyzed *A. pavonina* seed protein solution was loaded onto a column of preswollen microgranular DEAE cellulose (2.5 × 20 cm) previously equilibrated with 100 mM Tris-HCl, pH 8.0 buffer. The column was washed with 2 bed volumes of the same buffer and then subjected to a step gradient of 50, 100, 200, 250, 300, 350, 400, and 500 mM NaCl, prepared in 100 mM Tris-HCl, pH 8.0 buffer. Fractions of 5 mL were collected, and alternate fractions were monitored spectrophotometrically at 280 nm. Alternative DE-52 fractions were examined by SDS − PAGE and assayed for trypsin inhibitor activity. Four distinct protein fractions with trypsin inhibitor activity were separately pooled and dialyzed against 2 L of 100 mM Tris-HCl, pH 8.0 at 4 °C.

### 2.8 Effect of roasting and boiling and measurement of trypsin inhibitor activity

Five grams of seed material was used for studying the effect of roasting and boiling on trypsin inhibitor activity. Whole seeds (with seed coats) were roasted in a multifunctional heating and drying oven (Binder GmbH, Tuttlingen, Germany) at 120 °C for 1 h. For studying the effect of boiling, 5 g of seeds were placed in glass tubes and enough distilled water was added so that the seeds were immersed. Seed samples were boiled for 5, 10, 15, and 30 min and brought to room temperature. We also performed a parrel experiment where *A. pavonina* seeds without their seed coats were also subjected to roasting and boiling treatments. Roasted and boiled seed samples were frozen in liquid nitrogen, ground to a fine power with a mortar and pestle and used for measuring trypsin inhibitor activity.

Trypsin inhibitor activity assay was performed as described earlier (Xu, Qu, Song, Liu, Chen, & Krishnan, 2019). Briefly, *A. pavonina* seed powder (20 mg) was extracted with 1 mL of 10 mM NaOH for 15 min at room temperature. After centrifugation at 15800xg for 5 min, the clear supernatant was saved, and the protein concentration was determined by a colorimetric method. About 8–10 µg of seed protein was added to 1 mL (final volume) of assay buffer (50 mM Tris-HCl, 20 mM CaCl2, pH 8.2) containing 20 µg of trypsin and 1 mM α-*N*-benzoyl-dl-arginine-p-nitroanilide hydrochloride (BAPNA). The reaction was carried out for 10 min in a 37 °C incubator, terminated by the addition 500 μl of 30% acetic acid, and then the absorbance at 410 nm was recorded. Trypsin inhibitor activity (units/mg protein) was calculated from the absorbance read at 410 nm against a reagent blank. Trypsin units inhibited (TUI) was defined as the amount of inhibitor that reduces the absorbance of the noninhibited reaction by 0.1. ANOVA was performed and the data were corrected using square root (x + 0.1) to meet the assumptions, and means were compared in Fisher’s LSD with Tukey HSD adjustments using PROC GLIMMIX in SAS 9.4 (SAS Institute, Cary, NC), at p = 0.05.

### 2.9 Simulated gastric fluid (SGF) digestion stability of *A. pavonina* seed proteins

*Adenanthera pavonina* seed proteins were digested with pepsin (Sigma, St. Louis, MO) for different time periods at 37 °C in SGF (100 mM HCl, pH 1.5) following established protocol (Lee, & Hamaker, 2006). Briefly, 100 μg *A. pavonina* seed proteins were added to 100 μL aliquots of prewarmed SGF containing 0.03 mg/mL of pepsin in 1.5 mL microcentrifuge tubes. The pepsin digestion was performed in a 37 °C incubation water bath. The reactions were terminated at 0, 5, 15, 30, and 60 min after incubation by the addition of 10 μL of 1 N NaOH and 20 μL of 6X SDS-sample treatment buffer (350 mM Tris-HCl, 10% SDS, 30% glycerol, 15% (v/v) β-mercaptoethanol, and 175 mM bromophenol blue, pH 6.8) followed by boiling the samples for 5 min. Proteolytic fragments generated due to pepsin digestion were separated on 15% SDS-PAGE gels and visualized by staining the gels with Coomassie Blue.

### 2.10 Immunohistochemical localization and confocal microscopy

Seeds of *A. pavonina*, which were soaked overnight in sterile distilled water, were dissected into small pieces with a razor and immediately fixed in 50% ethyl alcohol, 5% glacial acetic acid and 10% formaldehyde for 24 h at 4 °C. The seed tissue was dehydrated sequentially in a graded ethanol/xylene series and infiltrated with paraffin as described earlier (Kim and Krishnan, 2004).

Immunohistochemical analysis was performed on paraffin sections using soybean trypsin inhibitor antibodies (Krishnan, 2001). Five-µm sections were mounted onto X-tra Plus microscope slides (Leica, Richmond, IL). Prior to immunohistochemical analysis sections were de-waxed in xylene, rehydrated through graded concentrations of ethanol, and finally in water. Slides were treated with 3% hydrogen peroxide (to inactivate endogenous peroxidase activity), and rinsed prior to incubation in blocking buffer with 5% bovine serum albumin for 20 min. Sections were then incubated for 60 min at room temperature with 1:200 dilution of a rabbit polyclonal soybean trypsin inhibitor antibody. Sections were then washed (DAKO, Carpenteria, CA), incubated for 30 min with EnVision, a horseradish peroxidase–labeled polymer conjugated to anti-rabbit antibodies (DAKO). Bound antibodies were visualized following incubation with 3,3′-diaminobenzidine solution (0.05% with 0.015% H_2_O_2_ in PBS; DAKO) for 3–5 min. Sections were counterstained with Meyer’s hematoxylin, dehydrated, and cover-slipped for microscopic examination. Images of stained sections were acquired with a Leica DM5500B widefield microscope (Leica Microsystems, Buffalo Grove, IL, USA) equipped with a Leica DFC290 color camera. For confocal immunofluorescence microscopy, paraffin sections were incubated with soybean trypsin inhibitor primary antibody followed by a 30 min incubation with Alexa Fluor 488 Plus-conjugated goat anti-rabbit secondary antibody (Invitrogen/ThermoFisher) diluted 1:500. Sections were coverslipped with a mounting medium containing an antifade and observed under a Leica SP8 laser scanning confocal microscope (Leica Microsystems, Buffalo Grove, IL, USA) with a 20x/NA 0.7 objective using a 488 nm excitation laser and a 500–550 nm bandpass.

### 2.11 Diet overlay toxicity assay in WCR larvae

Susceptibility of field-derived western corn rootworm larvae to *A. pavonina* trypsin inhibitors was examined by overlay on the insect diet. The eggs collected from the cages were kept in a dark chamber at 25–26 °C until bioassays started. When 50–100 neonate larvae were observed in the egg dishes, the eggs were washed and sterilized accordingly (Ludwick et al., 2018, Huynh et al., 2019). The assays were carried out in 96-well plates (Costar, Corning Incorporated, Corning, NY; Model # 3596), filled with 200 µL of WCRMO2 artificial diet (Huynh et al., 2019). Three different solutions of *A. pavonina* trypsin inhibitors diluted in TRIS buffer were used in the experiment, a crude extract (758.3 µg/cm^2^), DE-52 fraction A (189.5 µg/cm^2^), and DE-52 fraction B (151.7 µg/cm^2^). The buffers (10 mM Tris, pH 7.5, and 20 mM sodium citrate, pH 3.0) were used as negative controls and Gpp34/Tpp35Aa protein (former Cry34/35Ab1, 0.3 mg/ml (Cry34) and 1.0 mg/ml (Cry35) (provided by Genective Corp.) whole cell at 63.2 µg/cm^2^, diluted in sodium citrate buffer, was used as positive control. Twenty microliters of each treatment solution were dispensed per well and let dry in a flow hood until dry. One, less than 24 hr old, neonate was transferred to each well using a fine paint brush, plates were sealed with an adhesive sealer (Excel Scientific, Inc., Victorville, CA; Model # TSS-RTQ-100) and one hole was punched per well using a #0 insect pin. Larval mortality and the number of larvae that molted to 2nd and 3rd instars were recorded after 10 d and surviving larvae in each treatment were harvested in 0.5 mL centrifuge tubes containing 70% ethanol. The excess of ethanol was removed, and the tubes were placed in an oven (Blue M Therm Dry Bacteriological Incubator; Model # 602752) at 65 °C for at least 48 hr. Dry weight was recorded in a Sartorius Cubis ultra-micro scale (Sartorius Corporate, Göttingen, Germany; Model # MSU 6.6S-000-DM). The assays were run with five replicates, with eight larvae per treatment per replicate.

## 3 Results

### 3.1 Albumins are the predominant storage proteins of *A. pavonina* seeds

**Fig. 1:**
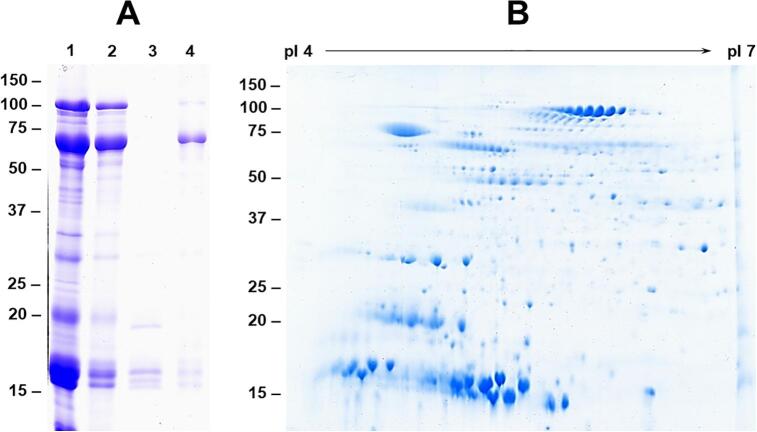
One and two-dimensional gel electrophoresis of *A. pavonina* seed proteins. Panel 1A. Seed proteins belonging to different solubility classes (albumin; lane 1, globulin; lane 2, prolamin; lane 3, and glutelin; lane 4) were fractionated by SDS-PAGE on a 13.5% gel. Panel 1B. Seed proteins (300 μg) were separated by isoelectric focusing on pI 4–7 strips, followed by SDS-PAGE on 15% gels. The gels were stained with Colloidal Coomassie Blue G-250. The position and sizes of protein markers in kDa are shown on the left side of the figures. (For interpretation of the references to color in this figure legend, the reader is referred to the web version of this article.)

Historically, seed proteins have been classified into albumins, globulins, prolamins and glutelins. Proteins that are soluble in water are termed albumins, in dilute saline as globulins, in alcohol water mixtures as prolamins and in dilute acid or alkali as glutelins (Shewry, Napier, & Tatham, 1995). To examine the abundance of different classes of seed proteins we first extracted *A. pavonina* seed powder with the different solutions and separated them by 1D SDS-PAGE (Fig. 1A. An examination of the protein composition of the albumin, globulin, prolamin and glutelin fractions clearly showed that proteins classified as albumins are the most abundant when compared to other classes of proteins (Fig. 1A. The albumin fraction was made up of several polypeptides whose molecular weights ranged from 120 kDa to 10 kDa. A protein with apparent molecular weight of 70 kDa was the most abundant followed by 17 kDa, 15 kDa and 100 kDa, respectively (Fig. 1A. A densitometer scan of the intensity of protein bands revealed that the 15–17 kDa bands account for about 27% of the total seed protein. Because of their abundance these proteins were also represented in the globulin fraction. Trace amounts of 17 kDa and 15 kDa proteins were also detected in prolamin fraction while a small amount of the 70 kDa protein was present in the glutelin fraction (Fig. 1A.

### 3.2 2-D gel electrophoresis reveals heterogeneity of *A. pavonina* seed proteins

To examine the complexity and heterogeneity of *A. pavonina* seed proteins, we resolved the extracted proteins by two-dimensional SDS-PAGE gel electrophoresis (Fig. 1B). Two-dimensional electrophoresis allowed the separation of *A. pavonina* proteins into more 200 discrete protein spots. The most abundant proteins seen in 1D SDS-PAGE gels (100 kDa, 70 kDa, 17 kDa, and 15 kDa) were resolved into numerous spots with distinct isoelectric points (Fig. 1B). For example, the 100 kDa protein was represented by series of protein spots whose isoelectric points ranged from 5.8 to 6.2. Similarly, the abundant 17 and 15 kDa proteins were also resolved into several distinct protein spots. The 17 kDa protein spots had more acidic isoelectric point than the 15 kDa protein spots. Additionally, several other protein spots with distinct isoelectric points were also detected.

### 3.3 Separation of *A. pavonina* seed proteins by DEAE anion-exchange chromatography

**Fig. 2:**
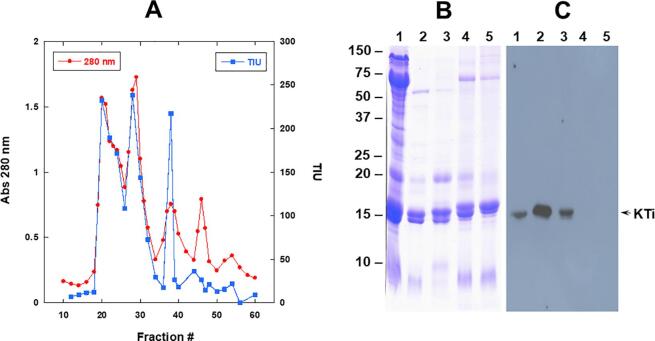
Separation of *A. pavonina* seed proteins by column chromatography and detection of trypsin inhibitors by immunoblot analysis. Panel A. A partially purified protein fraction was loaded on an ion-exchange DE-52 cellulose column. Unbound proteins were removed by washing the column with excess 100 mM Tris-HCl, pH 8.0 buffer. DE-52 cellulose bound proteins were eluted with a step gradient of sodium chloride (0–500 mM M) and 5 mL fractions were collected. Absorbance at 280 nm (0--0) and trypsin inhibitor activity (□----□) in alternate fractions were measured. Total seed protein and DE-52 peak A, B, C and D fractions were separated on a 15% SDS-PAGE gels. Resolved proteins were stained with Coomassie Blue (Panel B) or transferred to nitrocellulose membrane and incubated with soybean Kunitz trypsin inhibitor antibodies (Panel C). Immunoreactive proteins were detected by chemiluminescence using anti-rabbit IgG horseradish peroxidase conjugate. Lane 1, total protein; lane 2, DE-52 peak A; lane 3, DE-52 peak B; lane 4, DE-52 peak C; and lane 5, DE-52 peak D. The position and sizes of protein markers in kDa are shown on the left side of the figure. (For interpretation of the references to color in this figure legend, the reader is referred to the web version of this article.)

Previous studies have shown that seeds of *A. pavonina* contain high levels of trypsin inhibitor activity (Prabhu and Pattabiraman, 1980, Sowbaghya et al., 2019). To partially purify and characterize the trypsin inhibitors from the seeds of *A. pavonina* we initially fractionated the albumin protein fraction with CaCl_2_. We have previously shown CaCl_2_ can be utilized to obtain protein fractions that are enriched in trypsin inhibitors (Krishnan, Oehrlel, & Natarajan, 2009). This step resulted in the enrichment of abundant 17 and 15 kDa proteins which was subsequently fractionated on a DEAE cellulose column. Elution of the proteins bound to the DEAE cellulose with increasing salt concentration resulted in the separation of several distinct protein peaks (Fig. 2A). When alternative DE-52 fractions were assayed for trypsin inhibitor activity four distinct peaks of activity was detected (Fig. 2A). Protein fractions corresponding to the four peaks of trypsin inhibitor activity (Peak A, Peak B, Peak C, and Peak D) were pooled, and subjected to further biochemical analysis.

### 3.4 Soybean Kunitz trypsin inhibitor antibodies enables the identification of homologous *A. pavonina* seed proteins

**Fig. 3:**
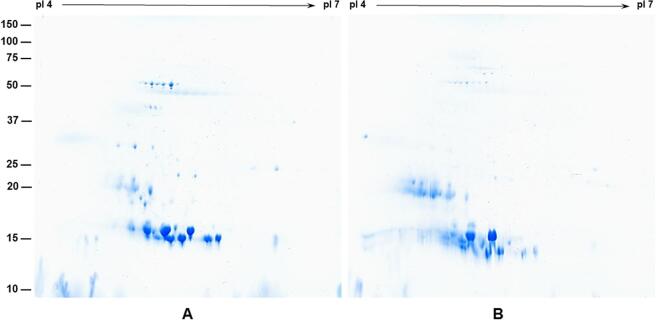
Two-dimensional gel electrophoresis of *A. pavonina* trypsin inhibitors. Proteins contained in DE-52 peak A (Panel A) and DE-52 peak B (Panel B) were individually separated by isoelectric focusing on IPG strips and then by SDS-PAGE on 13.5% gels. The gels were stained with Colloidal Coomassie Blue G-250. The position and sizes of protein markers in kDa are shown on the left side of the figures. (For interpretation of the references to color in this figure legend, the reader is referred to the web version of this article.)

**Supplementary Fig. 1:**
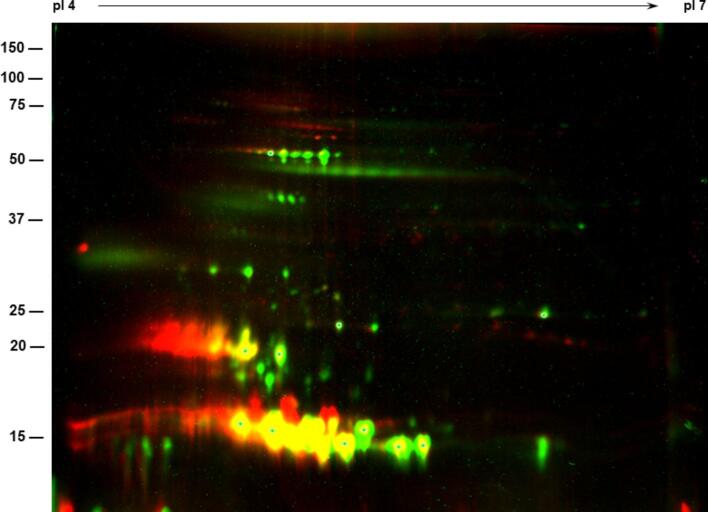
Overlay of 2D gels of DE-52 peak A and DE-52 peak B proteins using Delta2D software. Proteins contained in DE-52 peak A and DE-52 peak were individually separated by isoelectric focusing on IPG strips and then by SDS-PAGE on 13.5% gels. Images of Coomassie Blue stained gels were imported into Delta2D, assigned two different colors (green = DE-52 peak A; red = DE-52 peak A) and overlaid to provide a combined fusion image. The green color denotes those protein spots that are unique to DE-52 peak A, while red color denotes those protein spots that are unique to DE-52 peak B. Yellow demonstrates protein spots that are common to both DE-52 peak A and DE-52 peak B.

We analyzed the four DE-52 peaks containing trypsin inhibitor activity (A-D) along with the total seed proteins of *A. pavonina* seed by SDS-PAGE (Fig. 2B). An examination of the Coomassie stained gel, revealed DE-52 peaks A and B were enriched in 17 and 15 proteins, while DE-52 peaks C and D were enriched in 19 and 17 kDa proteins (Fig. 2B). To verify if these proteins are trypsin inhibitors, we performed immunoblot analysis using antibodies raised against purified soybean Kunitz trypsin inhibitor . Interestingly, soybean Kunitz trypsin inhibitor antibodies revealed cross-reactivity against the 15 kDa protein from DE-52 peaks A and B (Fig. 2C). Similarly, a weak cross-reacting protein was also detected in the total protein fraction. In contrast, no cross reaction was detected against DE-52 peaks C and D (Fig. 2C). To examine if the cross-reacting proteins in DE-52 peaks A and B are similar, we separated these DE-52 peaks by 2-D electrophoresis. Both DE-52 peak A and peak B were resolved into several spots with distinct isoelectric points (Fig. 3A and 3B). To visualize protein differences, we overlaid two identically resolved 2-D gels and analyzed the differences using Delta2D image analysis software (Supplementary Fig. 1. This examination clearly revealed the presence of several protein spots that are found in both but also heighted the presence of several unique protein spots. Our observation indicates that the *A. pavonina* seed trypsin inhibitors are a heterogenous group of proteins with distinct isoelectric points.

### 3.5 Effect of roasting and boiling on trypsin inhibitor activity

**Fig. 4:**
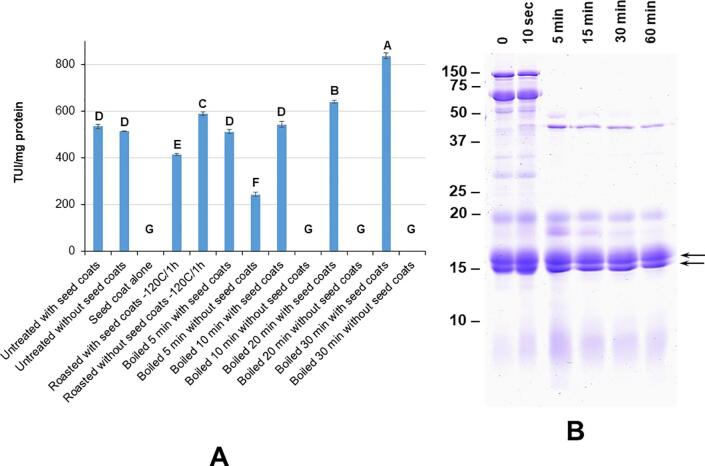
Effect of processing on trypsin inhibitor activity and digestive stability of *A. pavonina* seed proteins. Panel A. Trypsin inhibitor activity in unprocessed and processed seed samples are expressed as trypsin units inhibited (TIU)/mg protein. Trypsin units inhibited is defined as the amount of inhibitor that decreases the absorbance of the non-inhabited reaction by 0.1. The error bars indicate the standard error of the mean (n = 3). Different letters on the top of each column indicates significant differences between means. Panel B. Stability of *A. pavonina* seed proteins to pepsin digestion. Seed proteins were digested with pepsin over different time periods as indicated at the top of the figure. The protein products were fractionated on a 15% SDS-PAGE gel and stained with Coomassie Blue. The arrows points to the 15–19 kDa pepsin resistant proteins. (For interpretation of the references to color in this figure legend, the reader is referred to the web version of this article.)

Processing the seeds either by roasting or boiling have been shown to inactivate trypsin inhibitors from cereals and legumes. Hence, we evaluated the effect of roasting and boiling on trypsin inhibitor activity of *A. pavonina seeds*. Roasting whole *A. pavonina* seeds at 120 °C for 1 h lowered the trypsin inhibitor activity by about 22% when compared to unprocessed material (Fig. 4A. Interestingly, roasting marginally increased the trypsin inhibitor activity when the seed coats were removed before processing (Fig. 4A). Boiling the seeds with and without seed coats for various periods produced contrasting results. When the seed coats were retained, boiling enhanced the trypsin inhibitor activity in a time-dependent manner (Fig. 4A). In contrast, seeds devoid of seed coats lost trypsin inhibitor activity when boiled. About 54% reduction in trypsin inhibitor activity was seen within 5 min after boiling the seeds. Trypsin inhibitor activity was completely inactivated in seed samples that were boiled for 10, 20, and 30 min (Fig. 4A).

### 3.6 Abundant 15–17 kDa proteins of *A. pavonina* seed are resistant to pepsin digestion

One of the known benchmarks of food allergens is that they exhibit extraordinary digestive stability. Several food allergens are known to be stable when subjected to pepsin digestion (Goodman et al., 2008)). To evaluate the risk of the *A. pavonina* seed proteins as potential allergen, we investigated the ability of *A. pavonina* seed proteins to withstand pepsin digestion. (Fig. 4B). Apart from the 15–17 kDa proteins most other *A. pavonina* seed proteins were susceptible to pepsin digestion. Within 5 mins, most of the seed proteins were digested as indicated by the disappearance of these proteins after pepsin digestion (Fig. 4B). Concurrent with the disappearance of these major proteins several intermediate protein products were observed (Fig. 4B). In contrast, the 15–17 kDa proteins were unaffected and the relative amount of these protein bands remained almost unchanged even after 60 min incubation with pepsin (Fig. 4B).

### 3.7 Immunohistochemical localization of *A. pavonina* trypsin inhibitors

**Fig. 5:**
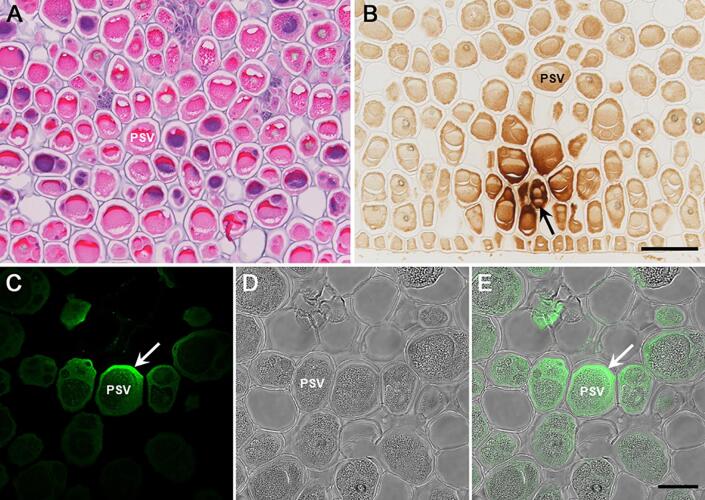
Immunohistochemical localization of *A. pavonina* trypsin inhibitors. Paraffin-embedded of *A. pavonina* seed was incubated with soybean trypsin inhibitor antibodies, followed by horseradish peroxidase–labeled polymer conjugated to anti-rabbit antibodies (DAKO). Bright-field view of hematoxylin-stained section of *A. pavonina* seed (Fig. 5A). Brown staining shows the location of specifically bound antibodies in the seed tissue (Fig. 5B, arrow). Immunofluorescence scanning confocal microscopy. Paraffin-embedded of *A. pavonina* seed was incubated with soybean trypsin inhibitor antibodies and goat anti-rabbit Alexa Fluor Plus 488 and observed by scanning confocal microscopy. Note green fluorescence signals mostly around the peripheral region of the cells (Fig. 5C, arrow). An overlay of bright-field (Fig. 5D) and fluorescence image (Fig. 5C) clearly shows the localization of trypsin inhibitors mostly in the cytosol of the cells (Fig. 5E, arow). PSV, protein storage vacuole. (For interpretation of the references to color in this figure legend, the reader is referred to the web version of this article.)

The abundance of *A. pavonina* trypsin inhibitors in the seeds and its cross-reactivity against soybean Kunitz trypsin inhibitor antibodies enabled us to investigate the localization of this protein. Immunohistochemical localization of trypsin inhibitors in *A. pavonina* seeds were performed by two different methods. In the first method, visualization of the antibody-antigen interaction was accomplished by chromogenic immunohistochemistry, wherein the secondary antibody was conjugated to horseradish peroxidase. In the second method, visualization of the antibody-antigen interaction was accomplished by immunofluorescence, where the secondary antibody was tagged to fluorescein. Examination of paraffin-embedded *A. pavonina* seed sections under bright-filed microscope revealed numerous densely packed parenchymatous cells. These cells contain a large central region, presumably protein storage vacuoles, which is surrounded by cytosol and other organelles (Fig. 5A). Observation of *A. pavonina* seed sections subjected to chromogenic immunohistochemistry revealed brown staining in all the cells (Fig. 5B). However, cells closer to the vascular bundles exhibited intense brown staining (Fig. 5B). Interestingly, the brown stain appears to be more specific to the cytosol than on the protein storage vacuoles (Fig. 5B).

To investigate the subcellular localization of trypsin inhibitors, we also performed confocal microscopy with immunofluorescent detection. This approach allowed a much clearer visualization of trypsin inhibitor localization in *A. pavonina* seed parenchymatous cells (Fig. 5C, 5E. Transmitted light scanning confocal microscopy clearly shows the presence of a prominent central region within each cell, which is surrounded by the cell cytosol (Fig. 5D. Confocal fluorescence microscopy observation of paraffin section challenged with trypsin inhibitor antibodies and goat anti-rabbit Alexa Fluor Plus 488 detected green fluorescence signals mostly around the peripheral region of the cells. No fluorescence signal was detected in the cell walls and the central granular region (protein storage vacuole) (Fig. 5C). Similarly, no specific signal was detected when the sections were incubated only with secondary fluorescent antibody. An overlay of transmitted light bright-field and fluorescence images clearly shows the localization of trypsin inhibitors mostly in the cytosol of the cells (Fig. 5E).

### 3.6 Susceptibility of field-derived western corn rootworm larvae to *A. pavonina* trypsin inhibitors

**Fig. 6:**
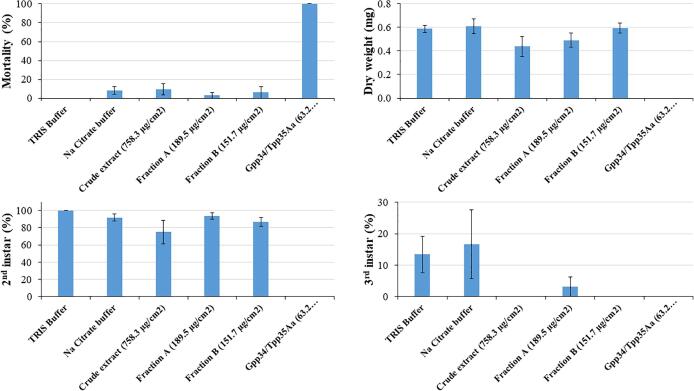
Mortality, larval dry weight, and percent of larvae that reached 2nd and 3rd instar after exposure of WCR larvae to *A. pavonina* trypsin inhibitors in different DE-52 column fractions, in diet overlay toxicity assays. Data points are the average (±SE) of five replicates with eight larvae per treatment per replicate.

**Supplementary Fig. 2:**
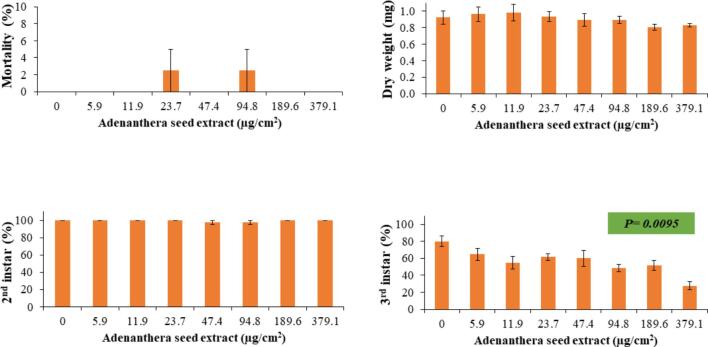
Mortality, larval dry weight, and percent of larvae that reached 2nd and 3rd instar after exposure of WCR larvae to various amounts of *A. pavonina* seed extract in diet overlay toxicity assays. Data points are the average (±SE) of five replicates with eight larvae per treatment per replicate.

Western corn rootworm (WCR)*, Diabrotica virgifera LeConte (Coleoptera: Chrysomelidae),* is one of the most devastating corn insect pests in the United States (Wechsler and Smith, 2018). WCR larvae cause significant damage to corn plants by feeding on the roots. Heavy infestation can significantly affect yield (Meinke et al., 2009). Annually, yield losses and control costs add up to an estimated $2 billion (Wechsler and Smith, 2018). This corn pest has evolved resistance to several control tactics including insecticides, crop rotation, *Bacillus thuringiensis* (Bt) proteins expressed in corn hybrids, and RNAi (Khajuria et al., 2018, Meinke et al., 2021). Earlier studies have shown that *A. pavonina* seed trypsin inhibitors could play an insecticidal function in the control of phytophagous insects (Macedo et al., 2002, Rodrigues Macedo et al., 2003, Macedo et al., 2010, Sasaki et al., 2015). Hence, we wanted to examine if the partially purified *A. pavonina* seed trypsin inhibitors have any insecticidal activity against western corn rootworm. This possibility was examined by exposing field-derived western corn rootworm larvae on a diet overlaid with *A. pavonina* seed trypsin inhibitors. Except for the positive control (Gpp34/Tpp35Aa), no significant differences were observed among the treatments for survival, dry weight, and 2nd and 3rd instar larvae (Fig. 5). However, numerically more larvae reached 2nd and 3rd instar in controls (buffer) when compared to the other treatments (Fig. 6). Gpp34/Tpp35Aa protein killed 100% of the WCR larvae as expected. In a preliminary assay, seven different concentrations (2-fold dilution from 5.9 to 379.1 µg/cm^2^) of *A. pavonina* crude seed extract plus buffer, were tested against WCR larvae; no significant differences were observed in survival, larval dry weight, and number of larvae that reached 2nd instar. However, significantly (F7,32 = 3.29; p = 0.0095) more larvae reached 3rd instar in buffer control when compared to the highest concentration at 379.1 µg/cm^2^ (Figure Supplementary Fig. 2).

## 4 Discussion

Like other legumes, *A. pavonina* seeds have a high protein content (Nwafor et al., 2017). One dimensional gel electrophoresis reveals that the most abundant seed proteins of *A. pavonina* have molecular weight of 100, 70, 17 and 15 kDa. Interestingly, these proteins were resolved by 2-D gel electrophoresis into a series of protein spots with similar molecular weights, potentially being encoded by multigene families or due to posttranslational modifications. Molecular cloning and sequencing of *A. pavonina* seed storage proteins should enable the identification of these proteins and their amino acid composition.

Legume seeds not only have high protein content but also accumulate significant amount of anti-nutritional factors such as trypsin inhibitors. Earlier work has shown that the seeds of *A. pavonina* contain very high levels of trypsin inhibitor activity (Prabhu and Pattabiraman, 1980, Sowbaghya et al., 2019). A comparison of trypsin inhibitor activity from several plant sources demonstrated that seeds of *A. pavonina* had the highest activity which was estimated to be about 3–16-times higher than that is present in soybean seeds (Prabhu and Pattabiraman, 1980, Sowbaghya et al., 2019). Several researchers have isolated and purified trypsin inhibitors from the seeds of *A. pavonina* (Prabhu and Pattabiraman, 1980, Richardson et al., 1986, Souza et al., 2016; Chandrashekharaiah, Shashank, Bharadwaj, Raju, Ramachandra Swamy, 2017). These studies have reported the presence of multiple isoinhibitors of trypsin in the seeds of *A. pavonina* (Richardson et al., 1986, Chandrashekharaiah et al., 2017). Three trypsin inhibitors named APPI-1, APPI-2and APPI-3 with an apparent molecular weight of 7–8 kDa, 11–12 kDa and 13–14 kDa have been purified from the seeds of *A. pavonina* by gel-permeation chromatography and RP-HPLC (Chandrashekharaiah et al., 2017). In contrast, eight isoinhibitors of trypsin (DE1-DE8) were isolated from *A. pavonina* seeds whose pI values ranged from 4.4 to 5.1. It was demonstrated that these isoinhibitors had a molecular weight of 21 kDa and was composed of alpha chain (16 kDa) and a beta chain (5 kDa) that was linked together by a disulfide bond. Amino acid sequence comparison of the one of the isoinhibitor (DE5) reveled 33–35% sequence homology with Kunitz-type trypsin inhibitors from soybean and winged bean (Richardson et al., 1986). In the present study, we have shown that *A. pavonina* trypsin inhibitors are composed of several distinct abundant protein spots with acidic isoelectric points. Unlike other studies, which have mainly focused on measuring the enzyme activity, our study has employed one and two-dimensional gel electrophoresis to establish the abundance of these proteins in *A. pavonina* seeds. In most legumes, trypsin inhibitors account for less than 5% of the total seed protein. In contrast, our study demonstrates that *A. pavonina* is unique since its seeds accumulate much higher amount of trypsin inhibitors accounting for nearly 27% of the total seed proteins. Additionally, our immunoblot analysis demonstrate that the soybean trypsin inhibitor antibodies cross-react against proteins present only in DE-52 peak A and B but not against proteins in DE-52 peak C and D. This observation is not surprising since *A. pavonina* trypsin inhibitor, a protein composed of two disulfide-linked polypeptide chains, shares only limited sequence homology with the single-chained soybean trypsin inhibitor. Soybean seeds contain at least three distinct trypsin inhibitors (Krishnan, 2001). The soybean trypsin inhibitor antibodies used in our analysis is a polyclonal antibody raised against the three different isoforms. It is likely that soybean trypsin antibodies are targeting different epitopes thus enabling the cross-reaction against only closely related *A. pavonina t*rypsin inhibitor isoforms.

The subcellular localization of trypsin inhibitor in legume seeds is debatable. Earlier, the subcellular localization of mung bean trypsin inhibitor was elucidated by cytochemistry with fluorescent antibodies. This study demonstrated that trypsin inhibitor was associated with the cytoplasm and not with the protein bodies where the most abundant seed proteins are stored (Chrispeels and Baumgartner, 1978). In contrast, an ultrastructural immunolocalization of Kunitz inhibitor trypsin inhibitor in soybean seed indicated its localization in multiple regions including cell walls, protein bodies, the cytoplasm, and the nucleus of the cotyledon and embryonic axis (Horisberger and Tacchini-Vonlanthen, 1983). In our study, by immunohistochemical localization we have clearly demonstrated that *A. pavonina* trypsin inhibitor is predominantly localized in the cytoplasm of the cells. Our observation in conjunction with the earlier report on mung bean strengthens the view that trypsin inhibitor is localized only in the cytoplasm.

Recently, attempts have been made to utilize milk made from *A. pavonina* seeds as an alternative source of protein (Afolabi et al., 2018, Dwitanti et al., 2020). Although the preliminary results are encouraging, the presence of high levels of trypsin inhibitor in *A. pavonina* seeds could pose a problem for its widespread use. The trypsin inhibitor activity in *A. pavonina* seeds is about 3–16-fold higher than that in soybean (Sowbaghya et al., 2019). Therefore, it will be critical to significantly lower or inactivate the trypsin inhibitor content of *A. pavonina* seeds to enhance its nutritional value. Our study demonstrates complete inactivation trypsin inhibitor activity from *A. pavonina* seeds is possible by boiling the seeds even for a short period. The seeds of *A. pavonina* are protected by a very hard seed coat and our study demonstrates that removal of the seed coat is essential for complete inactivation of the trypsin inhibitor. However, further investigation is required to elucidate the role of seed coat on influencing the trypsin inhibitor activity in *A. pavonina* seeds. We observed that roasting the seeds lacking seed coat marginally increased the trypsin inhibitor activity. On the other hand, boiling intact seeds caused a significant increase in trypsin inhibitor activity. It is possible that seed coats, which contain condensed tannins/polyphenols, may influence the trypsin inhibitor activity. We speculate that boing facilitates the release of tannins/polyphenols or other unidentified compounds into the surrounding water. Thus, in the absence of these inhibiting compounds, one could expect an increase in trypsin inhibitor activity. This possibility is strengthened by earlier studies which have shown that trypsin inhibitor activity can be inhibited by polyphenols or other inactivating factors (Chrispeels and Baumgartner, 1978, Ge et al., 2021). Additionally, the stability of the 15–17 kDa *A. pavonina* seed proteins to pepsin digestion also raises the allergenic potential of these abundant seed proteins. Previous studies have established that protein stability in SGF could be used as a reliable indicator of allergenicity. Clearly, additional studies are required to establish the allergenic potential of the 15–17 kDa *A. pavonina* seed proteins.

Paradoxically, the high trypsin inhibitor content of *A. pavonina* also functions as a valuable trait. Several studies have demonstrated that *A. pavonina* trypsin inhibitor possess antimicrobial, antifungal activity and can be utilized for biological control of insect pests (Macedo et al., 2002, Rodrigues Macedo et al., 2003, Macedo et al., 2010, Sasaki et al., 2015, Soares et al., 2012). When insect larvae were fed with artificial diets supplemented with *A. pavonina* trypsin inhibitors significant inhibition of the growth and development of the larvae was observed. This growth retardation has been attributed to the ability of trypsin inhibitors to bind and inactivate the insect digestive proteinases (Macedo et al., 2002). In our study, we did not observe significant effects of *A. pavonina* trypsin inhibitors on the growth and development of western corn rootworm larvae. Though numerically fewer WCR larvae reached 2nd and 3rd instar in presence of *A. pavonina* trypsin inhibitors, the overall effect was not as dramatic as reported against *Aedes aegypti* (yellow fever mosquito), *Anagasta kuehniella* (Mediterranean flour moth), *Anthonomus grandis* (bean weevil) and *Diatraea saccharalis* (sugarcane borer) (Macedo et al., 2002, Macedo et al., 2010, Sasaki et al., 2015). This is not unexpected, given that the insecticidal Bt toxins that have activity against WCR larvae are ineffective against lepidopteran pests and vice-versa.

## Funding

This research was supported by the Agricultural Research Service (Project Number: 5070–21000-040-00D), United States Department of Agriculture (USDA).

## CRediT authorship contribution statement

**Hari B. Krishnan:** Conceptualization, Formal analysis, Investigation, Resources, Writing – original draft. **Sunhyung Kim:** Investigation. **Adriano E. Pereira:** Investigation, Writing – review & editing. **Alexander Jurkevich:** Investigation, Writing – review & editing. **Bruce E. Hibbard:** Investigation, Resources, Writing – review & editing.

## Declaration of Competing Interest

The authors declare that they have no known competing financial interests or personal relationships that could have appeared to influence the work reported in this paper.
